# Bedside Availability of Prepared Oxytocin and Rapid Administration After Delivery to Prevent Postpartum Hemorrhage: An Observational Study in Karnataka, India

**DOI:** 10.9745/GHSP-D-14-00239

**Published:** 2015-06-12

**Authors:** Corrina Moucheraud, Jonathon Gass, Stuart Lipsitz, Jonathan Spector, Priya Agrawal, Lisa R Hirschhorn, Atul Gawande, Bhala Kodkany

**Affiliations:** ^a^​ Harvard T.H. Chan School of Public Health, Department of Global Health and Population, Boston, MA, USA; ^b^​ Ariadne Labs, Brigham and Women's Hospital and Harvard T.H. Chan School of Public Health, Boston, MA, USA; ^c^​ Brigham and Women's Hospital, Center for Surgery and Public Health, Boston, MA, USA; ^d^​ Massachusetts General Hospital, Boston, MA, USA; ^e^​ London School of Hygiene and Tropical Medicine, Faculty of Epidemiology and Population Health, London, UK; ^f^​ Harvard Medical School, Department of Global Health and Social Medicine, Boston, MA, USA; ^g^​ Jawaharlal Nehru Medical College, Women's and Children's Health Research Unit, Karnataka, India

## Abstract

Postpartum hemorrhage is a leading cause of maternal death worldwide. Rapid provision of uterotonics after childbirth is recommended to reduce the incidence and severity of postpartum hemorrhage. Data obtained through direct observation of childbirth practices, collected in a study of the World Health Organization’s Safe Childbirth Checklist in Karnataka, India, were used to measure if oxytocin prepared for administration and available at the bedside before birth was associated with decreased time to administration after birth. This was an observational study of provider behavior: data were obtained during a baseline assessment of health worker practices prior to introduction of the Safe Childbirth Checklist, representing behavior in the absence of any intervention. Analysis was based on 330 vaginal deliveries receiving oxytocin at any point postpartum. Oxytocin was prepared and available at bedside for approximately 39% of deliveries. We found that advance preparation and bedside availability of oxytocin was associated with increased likelihood of oxytocin administration within 1 minute after delivery (adjusted risk ratio = 4.89, 95% CI = 2.61, 9.16), as well as with decreased overall time to oxytocin administration after delivery (2.9 minutes sooner in adjusted models, 95% CI = -5.0, -0.9). Efforts to reduce postpartum hemorrhage should include recommendations and interventions to ensure advance preparation and bedside availability of oxytocin to facilitate prompt administration of the medicine after birth.

## Abstract (precis)

Advance preparation and bedside availability of oxytocin before childbirth was significantly and robustly associated with rapid administration of the utertonic, as recommended to prevent postpartum hemorrhage.

## INTRODUCTION

Worldwide, greater than a quarter million maternal deaths occur each year,^1^ and postpartum hemorrhage (PPH) is the leading direct cause.^2^ Incidence and severity of PPH can be reduced by active management of the third stage of labor (AMTSL), which includes early provision of uterotonics such as oxytocin.^3^^–^^5^

Oxytocin should be administered as quickly as possible after childbirth to prevent postpartum hemorrhage.

As a prophylactic intervention, it is essential that oxytocin be administered as quickly as possible after childbirth; current guidelines recommend administration of a uterotonic as part of AMTSL,^3^ and the International Confederation of Midwives (ICM) and International Federation of Gynaecology and Obstetrics (FIGO) recommend oxytocin administration within 1 minute of delivery.^4^ Unfortunately, many women globally do not receive this recommended preventive intervention.^6^ In addition, a multicountry study found that, even when oxytocin is given, there are errors in its administration, most commonly that administration is delayed beyond the recommended 1-minute postpartum time frame.^7^

Multiple studies have quantified poor rates of adherence to recommended AMTSL guidelines.^6^^–^^11^ In addition, qualitative research has explored possible causes of variation in use of AMTSL (including oxytocin administration), such as availability of supplies, lack of and low confidence in skills, and challenges in preparing and administering oxytocin rapidly after labor.^12^^–^^15^ However, little is known about factors associated with the timing of oxytocin use after delivery. In this investigation, we analyzed baseline observational data from a quality improvement study, of the Safe Childbirth Checklist (SCC) program of the World Health Organization (WHO), to explore whether preparing oxytocin injection prior to delivery and making it available at bedside was associated with increased likelihood of its rapid postpartum use in a hospital in India.

## METHODS

### Study Design

The SCC, developed by WHO, the Harvard T.H. Chan School of Public Health, and other partners, is designed to ensure health care providers’ adherence to 29 evidence-based essential birth practices in facility-based peripartum care. A pre-post study of the SCC in a sub-district hospital in Karnataka, India, found that an SCC-based intervention improved adherence to essential birth practices.^16^

### Data Collection

Our analysis uses data from the baseline period of this SCC study in Karnataka—prior to any intervention—during the observation period between delivery and 1-hour postpartum. Data for the study were collected via direct observation of health workers providing maternal and newborn care. Trained data collectors observed birth-related events during the intrapartum period, at pre-identified “pause points” between admission and discharge. Of relevance to this analysis, data collectors observed and recorded the timing of childbirth and of oxytocin administration.

### Data Analysis

We classified timing of oxytocin administration in one of three ways:

1. Within 1 minute of delivery (per ICM/FIGO recommendations)
2. Up to 2 minutes after delivery (broadened definition of rapid administration)
3. As a continuous value of time (minutes) between the birth event and postpartum oxytocin injection

Oxytocin was considered “prepared and available at the bedside” if it was drawn up into a syringe and available at the bedside antepartum, at the start of pushing.

The sample for this analysis was restricted to women with normal vaginal deliveries who received oxytocin at any point postpartum. Multivariate models were adjusted for delivery time (daytime [10:00-16:00] versus nighttime) and maternal risk factors for hemorrhage (age, parity, and long labor duration, defined as greater than 12 hours prior to admission for nulliparous women and greater than 24 hours for all other women).

Univariate analyses were performed using Rao-Scott chi-square tests.^17^ Generalized linear regression models, with a binomial distribution and a log link function, were used to examine whether oxytocin was administered within the recommended 1-minute time frame. For time to oxytocin administration as a continuous outcome, we used linear regression modeling.^18^ All models included standard errors clustered by provider. Analyses were conducted using Stata 12.1.

### Ethical Review

Study protocols were approved by ethics committees at Jawaharlal Nehru Medical College in Karnataka, WHO, the Harvard T.H. Chan School of Public Health, and the Health Ministry's Screening Committee, Indian Council of Medical Research.

## RESULTS

Our analyses were based on 330 deliveries. Oxytocin was prepared and available at bedside for 38.8% of deliveries (Table 1). Use of oxytocin within the recommended 1-minute time frame was higher when prepared oxytocin was available at bedside than when it was not prepared and available (15.6% of observed deliveries vs. 3.0%, respectively; *P*< .001). We found similar results when extending the time frame of administration: oxytocin was given within 2 minutes of delivery 43.8% of the time when it was prepared and available at bedside, compared with only 16.3% of the time when it was not (*P*< .001). The average time to oxytocin administration when oxytocin was prepared and available at bedside was 4.2 minutes—significantly shorter than the average time of 7.5 minutes when oxytocin was not prepared and at bedside (*P*< .001).

Oxytocin was prepared in advance and available at bedside for 39% of deliveries.

**TABLE 1.** Oxytocin Availability at Bedside and Time to Administration Among Vaginal Deliveries Receiving Postpartum Oxytocin, Karntaka, India (N = 330)

	**Prepared and Available at Bedside**	**Not Prepared and Not Available at Bedside**	***P*** **value**
No. (%)	128 (38.8)	202 (61.2)	
Received within 1 minute of delivery, No. (%)	20 (15.6)	6 (3.0)	< .001
Received within 2 minutes of delivery, No. (%)	56 (43.8)	33 (16.3)	< .001
Time to administration, mean (SD) [range], minutes	4.2 (4.6) [0–30]	7.5 (6.2) [0–30]	< .001
Time to administration, median (IQR [Q1-Q3]), minutes	3 (25 [0–25])	5 (29 [1–30])	< .001

Abbreviations: IQR, interquartile range; SD, standard deviation.

Regression models assessing the association between bedside availability and rapid oxytocin use suggest that deliveries in which oxytocin was prepared and available at bedside were nearly 5 times more likely than deliveries in which oxytocin was not available at bedside to have the medication administered within 1 minute postpartum, after adjusting for time of day and maternal risk factors for hemorrhage (adjusted risk ratio [RR] = 4.89, 95% confidence interval [CI] = 2.61, 9.16) (Table 2). When extending the time frame of administration to 2 minutes after childbirth, deliveries in which oxytocin had been prepared and available at bedside were 2.61 times more likely to have oxytocin administered than when it was not ready at the bedside (in the adjusted model, with covariates for time of day and maternal risk factors for hemorrhage) (95% CI = 1.26, 5.41). When time was examined as a continuous variable, deliveries in which oxytocin was prepared and available at bedside had oxytocin administered, on average, about 3 minutes sooner than deliveries in which oxytocin was not ready for administration at bedside, after adjusting for time of delivery and maternal hemorrhage risk factors (–2.9 minutes, 95% CI = –5.0, –0.9).

Advance preparation and bedside availability of oxytocin was significantly associated with administration within 1 minute of delivery.

**TABLE 2.** Association Between Time to Oxytocin Administration After Delivery and Bedside Availability of Oxytocin: Results From Unadjusted and Adjusted^a^ Regression Models Among Vaginal Deliveries Receiving Postpartum Oxytocin, Karnataka, India (N = 330)

	**RR (95% CI)**	**Adjusted RR (95% CI)**
Oxytocin administered within 1 minute	4.99 (2.53, 9.84)	4.89 (2.61, 9.16)
Oxytocin administered within 2 minutes	2.70 (1.38, 5.29)	2.61 (1.26, 5.41)
	**Minutes (95% CI)**	**Adjusted minutes (95% CI)**
Time to oxytocin administration, minutes	–3.3 (–5.2, –1.4)	–2.9 (–5.0, –0.9)

Abbreviations: CI, confidence interval; RR, risk ratio.

Data for oxytocin administered within 1 minute and 2 minutes report risk ratios from generalized linear models with a binomial distribution and a log link function; data for time to oxytocin administration report change in number of minutes, from linear regression models.

All results use standard errors clustered by provider.

a Adjusted for time of delivery (daytime/nighttime), mother’s age, parity, long labor (i.e., greater than 12 hours prior to admission for nulliparous women and greater than 24 hours for all other women).

## DISCUSSION

Advance preparation and bedside availability of oxytocin was associated with a significantly increased likelihood of its rapid use postpartum, when considering the ICM/FIGO-recommended 1-minute time frame as well as an expanded time frame of 2 minutes and time-to-use overall. Covariates representing time of day of the delivery and maternal risk factors for hemorrhage did not attenuate this effect.

Deliveries in which oxytocin was available at bedside received oxytocin, on average, about 3 minutes sooner than when there was no bedside oxytocin.

Those seeking to implement global guidelines for the prevention of postpartum hemorrhage may therefore want to consider adding operational recommendations to make oxytocin ready for administration and available at bedside—such as ensuring adequate supplies of the medication in the delivery room, preparing oxytocin syringes in advance, and assigning responsibility to guarantee these steps are routinely followed. The advent of prefilled oxytocin syringes (e.g., Oxytocin-in-Uniject devices) may facilitate rapid use after delivery.^15^^,^^19^ An important constraint is that oxytocin is not heat stable, although recent evidence from Ghana suggests that it may be shelf-stable around the point of care.^20^ So until additional evidence confirms the parameters of oxytocin’s stability, hospitals should seek to extend the cold chain into the labor ward (using an ice box, for example), so oxytocin can be stored close to the point of use rather than in general hospital drug storage. Behavioral prompts for providers may also be useful, such as keeping an empty needle and syringe near, or prepackaged in, the delivery kit. Finally, use of the SCC may help drive change, as results from the pilot study found that the intervention increased both pre-labor preparation of oxytocin and its prompt administration to women postpartum.^16^ Such changes could help increase the likelihood that health workers will deliver this potentially lifesaving intervention to all women in a timely manner.

Adding operational recommendations to ensure bedside availability of oxytocin at delivery may help prevent postpartum hemorrhage.

The findings from this study suggest that health system constraints and opportunities should be considered when developing global protocols. Medical practitioners may be responsible for implementing a number of different clinical guidelines—and policy makers should seek to understand how such guidelines are used in practice, and whether any operational challenges might be overcome with simple procedural solutions. We encourage future research on the barriers and enablers faced by health workers and health systems in implementing clinical guidelines to continue to drive improvement in obstetrics and beyond.

### Limitations

This study has some limitations. First, data were observational and collected at only 1 health facility—so we were able to measure only the association between bedside availability and timely use, rather than causality; and the findings may not be generalizable to all settings. Second, there is the possibility of measurement error. For example, without data on facility availability of oxytocin, the bedside availability variable represents both availability within the hospital and preparation at bedside. To explore the impact of possible measurement error in the independent variable, we dropped all births that occurred during time periods when bedside availability was at or below 10% for 3 days or longer—as a surrogate indicator of possible stock-outs—and reran the analyses. This restriction did not change the main results (likelihood of use within 1 minute of 3.45 [95% CI = 1.75, 6.81] when available at bedside in the fully adjusted model). Additionally, there were 3 women in the original dataset who had never received oxytocin postpartum; they were excluded from this analysis, but the near-universal administration of oxytocin at some point postpartum suggests that oxytocin was in stock at the facility at the time of all births studied here. Third, there is the possibility of omitted variable bias; however, the results were robust to the inclusion of covariates that represent risk factors for complications (which could theoretically be associated with need for medical care postpartum: maternal age, parity, and labor duration), although we cannot eliminate the possibility of confounding by unobserved variables. Lastly, this analysis did not assess health outcomes—so while we found that time to oxytocin administration was shorter when oxytocin was available and ready for administration at the bedside than when it was not, it was impossible to assess whether such improvements in practice could be expected to result in fewer cases of PPH and lower mortality.

## CONCLUSION

Adherence to global recommendations on preventing PPH through rapid administration of oxytocin was much higher when oxytocin was prepared and at bedside prior to delivery than when it was not. During the busy immediate postpartum period, under-resourced health systems with overtaxed health workers may have the additional need for such advance planning to enable rapid postpartum oxytocin administration—which also may have spillover effects to newborns, particularly those with urgent postpartum care needs. In resource-limited settings where a single health care worker must care for both mother and infant, advance preparation of oxytocin can facilitate rapid administration and thus avoid interfering with a health worker’s ability to promptly address the needs of the infant. Simple interventions, such as advance preparation of oxytocin and other essential birth supplies before delivery, are a core component of the SCC, and a study is underway to determine the impact of these and other critical components of care on maternal and neonatal morbidity and mortality.

## Notes

**Peer Reviewed**
